# A *CREB1* Gene Polymorphism (rs2253206) Is Associated with Prospective Memory in a Healthy Cohort

**DOI:** 10.3389/fnbeh.2017.00086

**Published:** 2017-05-16

**Authors:** Nesli Avgan, Heidi G. Sutherland, Rodney A. Lea, Lauren K. Spriggens, Larisa M. Haupt, David H. K. Shum, Lyn R. Griffiths

**Affiliations:** ^1^Genomics Research Centre, Chronic Disease and Ageing, Institute of Health and Biomedical Innovation, School of Biomedical Sciences, Queensland University of Technology, BrisbaneQLD, Australia; ^2^Menzies Health Institute Queensland and School of Applied Psychology, Griffith University, Gold CoastQLD, Australia

**Keywords:** CREB1, prospective memory, genetics of human memory, polymorphism, rs2253206

## Abstract

Prospective memory (PM) is generally defined as remembering to perform intended actions in the future and is important for functioning in daily life. Cyclic adenosine monophosphate (cAMP) responsive element binding protein 1 (CREB1) plays an important role in cognitive functions. In this study, we hypothesized that genetic variation in the *CREB1* gene is associated with PM. We genotyped a *CREB1* promoter polymorphism rs2253206 and tested it for association with PM in 619 healthy subjects. PM performance was measured using the Prospective and Retrospective Memory Questionnaire (PRMQ), the Comprehensive Assessment of Prospective Memory (CAPM), and the Memory for Intentions Screening Test (MIST). Generalized linear model analysis was conducted for each PM test independently using different inheritance models to identify any associations (*p* < 0.05). After multiple testing adjustments, a significant association was found between the rs2253206 genotype and PM performance for CAPM instrumental activities of daily living measure (*p* = 0.016). These results suggest that the rs2253206 polymorphism in the *CREB1* gene locus is associated with PM in healthy individuals and contributes to knowledge on the genetics of this particular type of memory.

## Introduction

Memory is a complex neurocognitive function, important in humans to establish an understanding of the past, present, and future (Kandel, 2006). In Squire’s widely accepted neuropsychological model of human memory, memory is divided into two subgroups: declarative and non-declarative memory. Declarative memory represents our conscious recall and is further divided into two subtypes, semantic and episodic memory (Squire, 1992). Episodic memory stores autobiographic events and semantic memory stores facts, concepts, and our knowledge (Tulving, 1985; Kandel, 2013). Collectively, these defined memory types refer to the past, and recollection of pieces of information from the past, further generalized under the term retrospective memory.

For remembering our intentions after a delay, we use prospective memory (PM). PM is essential for our understanding and sense of the future and refers to the function that enables a person to carry out a planned act after a delay (Burgess et al., 2001). Remembering to remember is the basic definition of PM and it can be divided into time- and event-based sub-types, and can also be triggered by cues (Baddeley et al., 2009; Burgess et al., 2011; Raskin et al., 2012). Remembering to call a friend at a certain time is an example of a time-based PM. Whereas if a friend asks you to give them a call when dinner is ready, remembering and calling the friend is an example of an event-based PM. Forgetting to call your friend at that required time, but remembering that you need to call them after seeing someone on the phone is an example for how PM can be triggered by a cue. One of the age-related memory impairments is PM failure, or forgetting intentions (Einstein et al., 2000). Dementia, Parkinson’s disease, Alzheimer’s disease, and schizophrenia also show PM impairments (Huppert and Beardsall, 1993; Jones et al., 2006; Woods et al., 2007; Kliegel et al., 2011). To date, little is known of the genetic factors which contribute to PM and its impairment, but as genetic variants have been shown to be associated with performance in a variety of memory subtypes, they are likely to also contribute to PM.

The cyclic adenosine monophosphate (cAMP) pathway has been shown to play an essential role in memory and cognitive abilities (Kandel, 2012). The cAMP responsive element binding protein 1 (*CREB1*) gene encodes a transcription factor that is a member of the leucine zipper family of DNA binding proteins (Quinn and Granner, 1990; Taylor et al., 1990). Studies have demonstrated the importance of CREB family proteins in learning and memory (Mizuno et al., 2002). CREB1 is a member of a family of proteins that function as transcription factors expressed in the brain (Kandel, 2012) with CREB signaling central to spatial, associative, emotional, and social memories in mammals (Gass et al., 1998; Pittenger et al., 2002; Viosca et al., 2009). Studies in mice and rats have described the role of CREB as a universal modulator of memory formation (Lazary et al., 2011). In humans, cognitive disorders and neurodegeneration disease studies on Huntington’s disease, Alzheimer’s disease, Rubinstein–Taybi syndrome, and Coffin–Lowry syndrome, support a key role for CREB signaling (Sweatt, 2009). Furthermore, CREB has been shown to play an essential role in synaptic plasticity, a feature of the pathways of memory formation (Kanehisa, 2002; Mantamadiotis et al., 2002; Saura and Valero, 2011).

Several studies focusing on depression, schizophrenia, rumination, and negative emotionally related memory impairments have found significant associations with CREB regulation (Kawanishi et al., 1999; Barrot et al., 2002; Lazary et al., 2011; Guo et al., 2014). Genetic polymorphisms can influence the expression of linked loci. Lazary et al. (2011) reported the involvement of rumination in memory and identified significant associations between the GG genotype of rs2253206 in *CREB1* and rumination and negative emotionality in two independent Caucasian cohorts (*n* = 651 and *n* = 1174). The rs2253206 polymorphism is located in the promoter region of *CREB1* gene (Sands and Palmer, 2008; Rankinen et al., 2010). Furthermore, Rankinen et al. (2010) revealed that the *CREB1* polymorphism rs2253206 major G allele is associated with significantly lower promoter activity than the minor A allele. Given these results we hypothesized that, as CREB1 plays an important role in cognition and memory, polymorphisms that influence *CREB1* expression may impact on memory performances of individuals.

In the present work, we conducted an association study focusing on the rs2253206 polymorphism in *CREB1* and PM performance. We investigated six different aspects of PM examined by three different memory tests in a quantitative manner, and report significant associations for some of these test scores with the *CREB1* rs2253206 polymorphism in a healthy cohort of individuals.

## Materials and Methods

### Study Participants

Individuals (*n* = 619) were recruited through advertisements at the Queensland University of Technology (QUT) and Griffith University campuses, with volunteers also invited to participate by posters and advertisements displayed around local shopping centers and health clinics for memory testing and subsequent genotyping. Participation was excluded for individuals with a history of psychiatric disorder or head injury to maintain a representative sample of cognitive and memory ability without additional complications. Due to the participation criteria, six individuals with a history of psychological background were excluded from the study and 12 additional participants were excluded after genotyping to maintain a stringent confidence interval (99%) in the data set. Thus, the study examined 601 healthy individuals (429 females and 172 males; median age: 20; range; 16–65 years.). Participants largely reported English to be their first language (89%), identified as mostly Australian (74%) in ethnicity and had varying education levels. All 601 participants met the study requirements of non-pathological healthy individuals. The study was approved by the Griffith University (MSC/01/09/HREC) and QUT Human Research and Ethics (1300000486) Committees. Written informed consent was provided by all participants prior to any study activities. Participants undertook a comprehensive battery of memory tests and completed self-report questionnaires to gauge their memory status. Two subsets (vocabulary and matrix reasoning) of The Wechsler Abbreviated Scale of Intelligence (WASI) were completed to estimate the intelligence quotient (IQ) of participants.

### Memory Testing for PM

We have employed three memory battery tests comprised of six memory subtests to assess memory performances of the participants, and utilized each score as independent phenotypes, since each measurement has different combination of elements. A relevant subset of questions (*n* = 8) in the Prospective and Retrospective Memory Questionnaire (PRMQ) was used to measure PM. PRMQ evaluates memory failures on a 5-point scale ranging from never to very often, concerning everyday life basis memory tasks and this questionnaire creates a three-dimensional result by combining long-term and short-term memory, and cue factors while assessing PM. An example for this test can be given as “Do you forget to buy something you planned to buy, like a birthday card, even when you see the shop?” (Smith et al., 2000; Crawford et al., 2003). For measuring PM capacity in addition to PRMQ, the Comprehensive Assessment of Prospective Memory (CAPM) test was also undertaken. This questionnaire has the same ranging scale as the PRMQ. It defines the frequency of PM failures in everyday life in regard to instrumental activities of daily living (IADL) and basic activities of daily living (BADL). Examples for IADL and BADL are “Leaving the iron on” or “Not locking the door when leaving home,” respectively (Chau et al., 2007). For the above tests, a higher score reflects more memory failures and therefore poorer PM. Furthermore, Memory for Intentions Screening Test (MIST) trials were administered to the participants to measure the execution of PM, including time and event based, with and without cues. The MIST has two sub-scores, the immediate MIST score (MIST-IMD) comprised of eight PM tasks which participants perform during the memory evaluation such as “When I show you a red pen, sign your name on the paper,” as well as a delay score (MIST-Delay), in which they were asked to email the examiner the next day at a certain time to address a query by the examiner (Woods et al., 2008; Kamat et al., 2014). For MIST, a higher score reflects better PM performance.

### Genotyping

Saliva samples were collected from each participant immediately after completion of the memory tests using Oragene^®^ DNA Self-Collection kits (DNA Genotek Inc., Ottawa, ON, Canada). DNA was extracted from whole saliva samples using the kit and protocol of the same manufacturer. The rs2253206 polymorphism in the *CREB1* gene was genotyped using the MassARRAY^®^ System by Agena Bioscience^TM^ (San Diego, CA, USA) on a 96-well MassARRAY platform. Amplification and extension primers used to detect the variant were designed with the Assay Design Suite (Agena Bioscience^TM^) and purchased from IDT (Singapore). The assay is based on the mass of the post-polymerase chain reaction (PCR) single base extension as previously described (Gabriel et al., 2009).

### Data Analysis

All descriptive statistics of the population and the phenotype data were carried out using The R Program for Statistical Computing (v3.2.2; R Development Core Team, 2015). Correlations between selected memory tests (PRMQ-PM, CAPM-IADL, CAPM-BADL, CAPM-Total, MIST-IMD, MIST-Delay) and IQ were investigated using Pearson’s *r* test. Quality control (QC) of the data and quantitative genetic analyses were conducted using PLINK (v1.09; Purcell et al., 2007). Due to the constructional differences of each assessment, we chose to treat memory scores individually as independent hypotheses and therefore did not adjust for multiple testing. Generalized linear model analysis for each memory phenotype individually was conducted using an additive model with a statistical significance level of *p* < 0.05 used to identify associations. Age, sex, and IQ were considered as covariates in the analysis. Analyses were also performed for dominant and recessive models of inheritance to compare the beta scores of all models to estimate genotypic effects on memory status. Consequently, to avoid false positive results we have accounted for multiple testing with respect to using three inheritance models and adjusted our *p*-value threshold using Bonferroni correction by setting an alpha-level of 0.0166 as the statistical significance threshold. Finally, we have further investigated the *CREB1* gene and the rs2253206 polymorphism using Genotype-Tissue Expression (GTEx) Browser^1^ to study the differences in tissue-specific levels by expression quantitative trait loci (eQTL) analysis (GTEx Consortium, 2013), and LDlink to explore the linkage disequilibrium (LD) between rs2253206 and the single nucleotide polymorphisms (SNPs) in close proxy, using all 1000 Genomes populations, to find putatively functional variants in our region of interest (Machiela and Chanock, 2015).

## Results

In this study, PM performance was measured using three memory tests widely used to assess memory impairments (Raskin et al., 2012): PRMQ, CAPM, and MIST. PRMQ and CAPM are self-report questionnaires. PRMQ evaluates RM and PM with equal number of questions, but in this study only the aspect that assesses PM was used, which focuses on common everyday-life memory failures such as forgetting to take a pill (Smith et al., 2000). CAPM assesses two different PM failures; basic daily routine failures such as forgetting to eat a meal or forgetting to put on a piece of clothing (e.g., socks) are measured using BADL, and instrumental activity related failures such as leaving the stove on or having trouble remembering personal dates at the right time (e.g., birthdays) are measured using IADL (Chau et al., 2007). The MIST is a one-on-one task-based test measuring time- and event-based planned acts with and without cues, using eight different PM tasks while solving a word-search puzzle as a distraction (Kamat et al., 2014). Examples of MIST for PM assessment include: “In exactly 15 min please tell me it is time to take a break” for time based; and “When I hand you a red pen, please sign your name on your paper” for event based with a cue. MIST also includes a delay test in which participants were asked to send an email to the examiner at a certain time, answering a specific question asked during the test (Woods et al., 2008; Kamat et al., 2014). We have studied these memory measurements independently due to the distinct elements of each assessment.

### Descriptive Statistics of the Memory Cohort

As a result of the SNP array QC step, 12 individuals were excluded from further analyses due to poor genotyping quality. Genetic analysis was performed on 601 healthy individuals with IQ normally distributed in the samples, ranging from 78 to 138 with a mean IQ of 108 (SD = 10.77). Demographics of the memory cohort are presented in **Table 1**. Two-thirds of the cohort was female (71%). The age of participants ranged from 16 to 65 years and the majority of the cohort were Australian. With respect to education determined by the highest qualification, the majority of the individuals in the cohort were high school graduates, but a large number were continuing to a diploma or a bachelor’s degree due to the high number of individuals in the younger age class (16–25 years) present in the cohort.

**Table 1:** Demographics of the Genomics Research Centre (GRC) memory cohort.

Variable	Participants (*n* = 601), *N* (%)
**Age group**	
16–25	469 (77.04)
26–35	79 (13.14)
36–45	36 (6.00)
46–55	10 (1.66)
56–65	7 (1.16)
**Gender**	
Male	172 (28.62)
Female	429 (71.38)
**Ethnicity**	
Australian	446 (74.88)
Other	155 (25.12)
**Highest qualification**	
School certificate	376 (62.56)
Diploma program	143 (23.79)
Bachelor’s degree	59 (9.82)
Postgraduate degree	21 (3.49)
Other	2 (0.33)

Correlation analysis showed that IQ was not correlated with any of the memory tests. When we look further into subtests of CAPM, CAPM-Total was strongly correlated within CAPM-IADL (*r* = 0.83) and CAPM-BADL (*r* = 0.79), which is expected because the CAPM-Total is made up of these two sub-scores. Additionally, the PRMQ and CAPM-Total were moderately correlated with each other (*r* = 0.58). This may reflect the nature of both tests; both are self-answered questionnaires focused on daily memory failures, yet they do have some constructive differences. In contrast, despite the use of MIST as a PM measure we did not find it to be correlated with either PRMQ or CAPM. Of note, MIST is not a questionnaire but a memory test, administered by the examiner and requires behavioral responses.

### Genetic Association Analysis

The rs2253206 SNP passed QC and genotype distribution was found to be in Hardy–Weinberg equilibrium (>0.05). Genetic association analysis was then completed using age, gender, and IQ as covariates, to focus solely on the effect of the polymorphism on PM performance. Recessive, additive, and dominant models of inheritance were undertaken with linear regression analysis to consider possible allelic affects and to account for multiple testing for these models we have adjusted the significance threshold for this study by using Bonferroni correction, setting the significance *p*-value to 0.0167. Results are summarized in **Table 2**. A significant association was identified between the rs2253206 variant and the CAPM-IADL memory phenotype in the additive model [*p*_CAPM(IADL)_ = 0.016]. Additionally, we found nominal associations for CAPM-IADL in the recessive model [*p*_CAPM(IADL)_ = 0.033] and the MIST-IMD memory phenotype in the dominant model [*p*_MIST(IMD)_ = 0.039].

**Table 2:** *CREB1* variant rs2253206 association with memory phenotypes for recessive, additive and dominant model of inheritance; controlling for IQ, gender, and age.

Memory test	Assessed memory type	Recessive model			Additive model			Dominant model		
		β	*t*	*p*	β	*t*	*p*	β	*t*	*p*
CAPM	Prospective memory									
	IADL	–0.124	–2.141	**0.033**	–0.079	–2.424	**0.016**	–0.091	–1.838	0.067
	BADL	–0.039	–0.840	0.401	–0.033	–1.260	0.208	–0.046	–1.190	0.235
	Total	–0.052	–1.161	0.246	–0.041	–1.624	0.105	–0.056	–1.466	0.143
MIST	Prospective memory									
	Immediate	0.059	0.371	0.711	0.141	1.576	0.116	0.280	2.068	**0.039**
	Delay	1.164	1.238	0.216	0.861	1.630	0.104	1.125	1.409	0.159
PRMQ	Prospective memory	–0.624	–1.309	0.191	–0.425	–1.588	0.113	–0.520	1.285	0.199

*CREB1, cyclic adenosine monophosphate responsive element binding protein 1; CAPM, Comprehensive Assessment of Prospective Memory; IADL, instrumental activities of daily living; BADL, basic activities of daily living; MIST, Memory for Intentions Screening Test; PRMQ, Prospective and Retrospective Memory Questionnaire. Bolded values shows the p < 0.05.*

The frequency of the minor A-allele for the rs2253206 polymorphism in our cohort was calculated to be 0.437, with genotype distributions of 19.1% for AA, 49.6% for AG, and 31.3% for GG genotypes (**Table 3**). Individuals carrying the A-allele showed a stronger association with the CAPM-IADL test results in the additive model of inheritance compared with the recessive model of inheritance (*p* = 0.016 vs. *p* = 0.033). In the CAPM test, a higher score reflects poorer memory performance and **Table 3** shows that the A-allele is associated with lower CAPM-IADL memory test scores, corresponding to less memory failures and a better PM performance. In the MIST, a higher score reflects better memory performance and **Table 3** shows that for MIST-IMD the A-allele was correlated with higher test scores. Therefore, the significant results of CAPM-IADL (additive model) and nominally significant results of MIST-IMD (dominant model) in the same population are consistent with the minor A-allele of rs2253206 being correlated with better PM performance. Furthermore, the direction of the beta score obtained for the MIST-IMD association analysis was opposite that obtained for the CAPM-IADL test as would be expected by this suggestion (**Table 2**). In the PRMQ-PM test higher test scores reflect poorer memory performance. While the results for PRMQ-PM were not significant, the A-allele was also correlated with better PM performance, as well as higher IQ scores (**Table 3**).

**Table 3:** *CREB1* variant rs2253206 allelic distribution and allele counts, with memory and IQ score distributions.

Test	Assessed memory type	AA (*n* = 115)	AG (*n* = 297)	GG (*n* = 189)
		Mean (min–max)	Mean (min–max)	Mean (min–max)
CAPM	Prospective memory			
	IADL	2.048 (1.00–3.88)	2.143 (1.00–4.43)	2.224 (1.13–4.00)
	BADL	1.515 (1.00–3.25)	1.540 (1.00–3.25)	1.581 (1.00–3.13)
	Total	1.796 (1.062–3.13)	1.828 (1.00–3.27)	1.885 (1.13–3.5)
MIST	Prospective memory			
	Immediate	14.700 (5.00–16.00)	14.740 (9.00–16.00)	14.440 (6.00–16.00)
	Delay	1.365 (0.00–2.00)	1.279 (0.00–2.00)	1.185 (0.00–2.00)
PRMQ	Prospective memory	20.380 (10.00–38.00)	20.820 (12.00–38.00)	21.330 (11.00–37.00)
IQ		108.522 (80–130)	108.250 (78–138)	107.598 (78–134)

*CREB1, cyclic adenosine monophosphate responsive element binding protein 1; CAPM, Comprehensive Assessment of Prospective Memory; IADL, instrumental activities of daily living; BADL, basic activities of daily living; MIST, Memory for Intentions Screening Test; PRMQ, Prospective and Retrospective Memory Questionnaire; IQ, Intelligence Quotient.*

**FIGURE 1:**
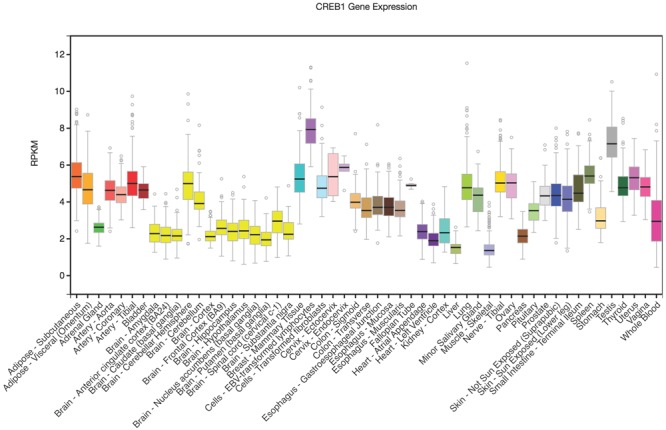
***CREB1* differential gene expression illustrated across tissues.** Vertical axis is RPKM (reads per kilobase transcript per million) and horizontal axis shows the tissues. GTEx Browser (http://www.gtexportal.org/home/). The Genotype-Tissue Expression (GTEx) Project was supported by the Common Fund of the Office of the Director of the National Institutes of Health, and by NCI, NHGRI, NHLBI, NIDA, NIMH, and NINDS. The data used for the analyses described in this manuscript were obtained from: the GTEx Portal on 04/07/17.

Rankinen et al. (2010) reported that the minor A-allele of rs2253206 was associated with lower *CREB1* promoter activity compared to the G-allele in transfected mouse skeletal muscle cell lines. In order to further explore how rs2253206 might affect *CREB1* expression in areas of the brain that are involved in memory processes we used the GTEx eQTL Browser to investigate *CREB1* mRNA levels. *CREB1* is expressed in brain-related tissues (shown in yellow) and most highly in the cerebellum, but is at lower levels than some tissues where it is highly expressed such as testis (shown in gray) and transformed lymphocytes (shown in purple) (**Figure 1**).

**FIGURE 2:**
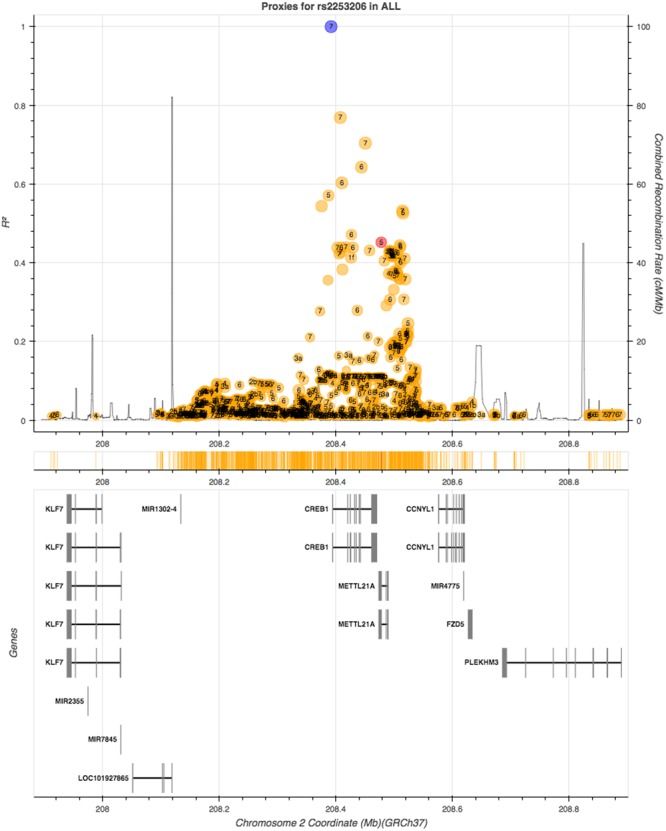
**Linkage disequilibrium (LD) proxy plot (generated using LDlink) for *CREB1* gene rs2253206 polymorphism.** Vertical axis is the LD (*r*^2^) measure and the horizontal axis is the chromosomal position with corresponding genes illustrated below. The blue dot represents rs2253206, yellow and red dots represent non-coding and coding region variants, respectively, and each variant has calculated for regulatory potential (1 being high and 7 being low).

We then queried GTEx for evidence of eQTLs of rs2253206. *CREB1* was not reported as an eQTL of the rs2253206 polymorphism in GTEx, however, a gene that overlaps with *CREB1*, methyltransferase like 21A (*METTL21A*), and a downstream gene, cyclin Y-like 1 (*CCNYL1*), are eQTLs for rs2253206 in some non-neural tissues. To determine whether any SNPs in high LD with rs2253206 may be influencing either *CREB1* expression levels or its protein we looked for SNPs that are in close proximity to rs2253206 polymorphism using LDlink, an online tool to investigate LD in population groups for a polymorphism or loci (Machiela and Chanock, 2015). Using 1000 Genomes data (phase 3) with all the populations, we could not identify any other polymorphism or functional variants in high LD (*r*^2^ > 0.8) with rs2253206 that could have provided further information to support explaining the effect of the rs2253206 polymorphism (**Figure 2**).

## Discussion

In this study, we used a non-pathological population-based sample of individuals to investigate the effect of the rs2253206 *CREB1* gene polymorphism on PM. After multiple testing corrections, we found that the minor A-allele at rs2253206 was significantly associated with better PM performance in an additive model of inheritance and the homozygous AA genotype was nominally associated in a recessive model of inheritance. In contrast, the homozygous major G allele was shown to be correlated with poorer PM and IQ scores (**Tables 2, 3**). The difference between the memory test scores for the three genotypes (AA, AG, and GG) are small and this is likely to due to the fact that the cohort is comprised of healthy, and mainly young, individuals. Nevertheless, small increments in the scores obtained in the PRMQ, CAPM, and MIST can reflect a large impact on memory performance and daily living. While these tests are commonly used to assess elderly individuals or those that might be affected by Alzheimer’s disease, Parkinson’s disease, schizophrenia, etc., our results suggest that genetic factors may contribute to performance of PM tasks in healthy individuals.

Two of the memory measures used in this study, PRMQ-PM and CAPM-BADL, are very similar in the questions asked, yet they are not highly correlated due to the different construction of the tests. Neither test was found to be significantly associated with rs2253206 in the study cohort. MIST-IMD, which evaluates PM behavioral performance with respect to immediate memory, was nominally associated with rs2253206, while MIST-Delay which measures another aspect of PM encompassing long-term memory, did not show any association. We also found that MIST-IMD and MIST-Delay test scores were not correlated with each other. All tests assess measures of PM, but lack of correlation between test scores suggest that it is a heterogeneous construct.

Several studies have demonstrated that CREB is essential for long-term memory and synaptic plasticity (Impey et al., 1996; Lamprecht et al., 1997; Josselyn and Nguyen, 2005; Alberini, 2009; Wallace et al., 2009). Additionally, Williams et al. (2008) have shown changing expression levels of CREB influences working memory in a rat model. SNPs in the *CREB1* gene, including rs2253206, have previously been significantly associated with traits linked to cognitive vulnerability. Guo et al. (2014) investigated the effect of *CREB1* gene polymorphisms on cognitive dysfunction in Chinese patients with major depression and their results suggest the *CREB1* gene is a promising marker for cognitive function in major depression patients. Juhasz et al. (2011) investigated risk factors for depression, and while studying the neuroplastic pathway, they demonstrated a significant association between the *CREB1* rs2253206 polymorphism with rumination and severe depression, reporting that minor allele carriers are less likely to ruminate. These results suggest that *CREB1* rs2253206 is, or is linked with, a functional SNP. However, we were unable to find evidence that *CREB1* is an eQTL of rs2253206 using the GTEx database, although it suggested that the nearby genes *METTL21A* and *CCNYL1* were in some non-neuronal tissues. Little is known of the function of these genes. Furthermore, we could not detect any other polymorphisms in high LD with rs2253206 that suggested a role in *CREB1* expression or function.

Functional analysis of the rs2253206 SNP by Rankinen et al. (2010) demonstrated that the major G-allele has lower promoter activity, likely due to loss of a CCAAT enhancer-binding protein alpha (C/EBPα) transcription factor binding site. Therefore, the effect of rs2253206 on cognitive function observed in our cohort may occur via altered expression levels of *CREB1*. While analysis of *CREB1* expression using GTEx did not show high levels in brain-related tissues, *CREB1* transcripts were present in all neural tissues and highest in cerebellar tissues. Although that part of the brain has not been reported to be particularly associated with PM, Tomlinson et al. (2014) found contribution of the cerebellum in working memory.

A limitation for our study is the small sample size which impacts on the power to find significant associations. Nevertheless, our findings suggest further investigation of the CREB pathway in our memory cohort may be justified, and also that the rs2253206 polymorphism should be tested in a similarly characterized independent cohort. However, with respect to PM few studies have investigated the genetic factors involved and thus have both PM phenotypes and genotyping data available. We have previously reported a significant effect of the *APOE* 𝜀4 polymorphism in PM using the CAPM test in a smaller subset of the cohort investigated here (Donges et al., 2012). Evans et al. (2014) also reported an association of *APOE* 𝜀4 genotype with PM using different PM tasks to our study; interestingly they found that genotype affects changed with age of the participants (Evans et al., 2014). Kennedy et al. (2015) investigating the brain-derived neurotrophic factor (*BDNF*) Val66Met polymorphism (rs6265) also found an adverse effect of age on PM performance across the lifespan which was much stronger in the *BDNF* Met carriers than for the Val homozygotes. Therefore, it would be of interest to also study the *CREB1* rs2253206 polymorphism in an older cohort tested for PM performance, when impairments influenced by genetic factors may become more pronounced.

In summary, we have identified associations between *CREB1* rs2253206 and PM in a healthy cohort; one of the memory phenotypes (CAPM-IADL) was identified as being significantly associated and another (MIST-IMD) identified as nominally associated with rs2253206. When we consider its location and evidence for an effect of the rs2253206 polymorphism on *CREB1* promoter activity, it is conceivable that genotypic variation may affect PM performance via a regulatory effect on the *CREB1* gene, a gene known to be involved in cognition and re-modeling of synaptic plasticity. Our results suggest that further studies of polymorphisms in *CREB1* and other genes in the cAMP pathway would be of interest to explore its role in the genetic basis of PM.

## Author Contributions

NA performed the laboratory work, data analysis, and interpretation. HS and RL supervised the study and interpretation of the results. DS designed the memory performance evaluations. LS participated in phenotype data collection. LH and LG supervised the study. All authors contributed to writing and editing of the manuscript.

## Conflict of Interest Statement

The authors declare that the research was conducted in the absence of any commercial or financial relationships that could be construed as a potential conflict of interest.

## Footnotes

1 http://www.gtexportal.org/home/
